# Antiangiogenic Treatment of Patients with Hereditary Hemorrhagic Telangiectasia: Experience of a Hungarian Center

**DOI:** 10.3390/jcm14228160

**Published:** 2025-11-18

**Authors:** Boglárka Brugós, Angéla Csirmaz, Tamás Major, Zsuzsanna Bereczky, Réka Gindele, Gábor Balogh, Sándor Kacska, Péter Sipos, Árpád Illés, György Pfliegler

**Affiliations:** 1 Division of Hematology, Faculty of Medicine, University of Debrecen, Nagyerdei krt. 98, 4032 Debrecen, Hungary; csirmaz.angela@med.unideb.hu (A.C.); illes.arpad@med.unideb.hu (Á.I.); 2 Division of Otorhinolaryngology, Head and Neck Surgery, Markhot Ferenc Teaching Hospital, 3300 Eger, Hungary; major.tamas@mfkh.hu; 3 Division of Clinical Laboratory Science, Faculty of Medicine, University of Debrecen, 4032 Debrecen, Hungary; zsbereczky@med.unideb.hu (Z.B.); gindele.reka@med.unideb.hu (R.G.); balogh.gabor@med.unideb.hu (G.B.); 4 Department of Gastroenterology, Faculty of Medicine, University of Debrecen, Nagyerdei krt. 98, 4032 Debrecen, Hungary; kacska.sandor@med.unideb.hu; 5 Faculty of Agricultural and Food Sciences and Environmental Management, Institute of Nutrition, University of Debrecen, 4032 Debrecen, Hungary; siposp@agr.unideb.hu; 6 Center of Expertise of Rare Diseases, University of Debrecen, 4032 Debrecen, Hungary; pfliegler@med.unideb.hu

**Keywords:** HHT, epistaxis, telangiectasia, thalidomide, bevacizumab

## Abstract

**Background:** Hereditary hemorrhagic telangiectasia (HHT) is an inherited vascular bleeding disorder. The most common symptoms are recurrent, severe nosebleeds that occasionally necessitate intervention by an ENT (Ear, Nose, and Throat) specialist, as well as iron-deficiency anemia. Telangiectasia is typically located in the nasal cavity, lips, tongue, fingertips, and the gastrointestinal mucosa. Arteriovenous malformations (AVMs) are located in internal organs (brain, lungs, liver, etc.). The family history is positive for HHT. The diagnosis is based on the Curacao criteria. The endoglin and activin receptor-like kinase 1 genes (*ENG* and *ACVRL1*) are the most common mutation sites, leading to elevated endothelial growth factor (VEGF) levels. **Methods:** We conducted a retrospective analysis in the Department of Internal Medicine, Division of Hematology, and Center of Expertise for Rare Diseases at the University of Debrecen, spanning the period from 2010 to 2025. Records of patients referred with HHT were reviewed concerning demographic data, clinical presentations, laboratory findings, and treatment approaches. To evaluate management options, epistaxis severity was assessed using the Epistaxis Severity Score (ESS). **Results:** 48 HHT patients (21 male and 27 female) were included in this retrospective study. Genetic testing was positive in each case, showing mutations in the *ENG* (HHT1 subgroup) or *ACVRL1* (HHT2 subgroup) genes. Most of the patients are followed-up with in our department. ESS was calculated at baseline and 6 months after antiangiogenic treatment by two independent physicians. Detailed computed tomography (CT) was performed in all patients. Seven patients were administered desmopressin, a synthetic analog of antidiuretic hormone (ADH), based on our previous experience in reducing bleeding in von Willebrand disease. Antiangiogenic therapy with thalidomide (50 mg oral tablets) was used in 24 patients, while bevacizumab was administered to 5 patients. Most patients experienced a remarkable decrease in epistaxis severity and a reduction in the need for transfusions (ESS before treatment: HHT1 patients, 4.15 ± 1.91 vs. ESS after treatment, 2.62 ± 0.99; HHT2 patients, 3.79 ± 3.19 vs. 2.02 ± 1.91). Subgroup analysis using paired ESS data showed a significant reduction in ESS in both HHT1 and HHT2 patients (*p* = 0.003 and *p* = 0.043, respectively). Bevacizumab further reduced the ESS, but the few cases were not suitable for statistical analysis. Serum iron levels significantly increased after antiangiogenic treatment in the HHT2 group (*p* = 0.01). **Conclusions:** HHT is a rare vascular bleeding disorder. Daily nosebleeds impair the patients’ quality of life and sometimes lead to severe transfusion-dependent iron-deficient anemia. Frequent hospitalization places a significant burden on the healthcare system. Thus, we have used treatment options for HHT patients that primarily act by inhibiting VEGF, and these treatment modalities have yielded successful results in our hands.

## 1. Introduction

Hereditary hemorrhagic telangiectasia (HHT), also known as Osler-Weber-Rendu disease, is named after the physicians who independently first described the syndrome at the end of the 19th and at the beginning of the 20th century [1,2]. The disease has autosomal dominant inheritance; it occurs in 1:5000–8000 individuals and affects both genders [3,4].

The diagnosis is based on the Curacao criteria [5]. It is definite if three or more criteria are present: 1, recurrent spontaneous epistaxis, 2, multiple telangiectasia at characteristic sites (lips, oral cavity, fingers, nose), 3, visceral lesions (gastrointestinal telangiectasia, pulmonary, hepatic, cerebral, spinal arteriovenous malformations (AVM)), 4, family history is positive (first-degree relative with HHT). The most common clinically significant complication is bleeding, and as a consequence, the development of iron-deficient anemia, which represents a considerable burden on patient care. Molecular testing is also available in HHT. The background of the disease is loss-of-function mutations in the genes encoding the endoglin and activin A receptor type II-like 1 (ALK1) proteins (*ENG* and *ACVRL1*, accounting for HHT1 and HHT2 phenotypes, respectively), which are detected in almost 90% of all cases [6,7]. The genes mentioned above and their products contribute to the activation of the transforming growth factor β (TGF-β)/bone morphogenetic protein (BMP) signaling pathway, which plays a significant role in angiogenesis. Other genes with mutations contributing to some cases of HHT include MADH4, which encodes SMAD4 (leading to the juvenile polyposis-HHT phenotype), and GDF2, which encodes BMP9, a ligand of ALK1 (HHT5 phenotype) [2,8,9]. Other rare phenotypes, such as HHT3 (5q31) [2,7,10,11,12] and HHT4 (7q14) [2,7,10,11,12], have also been reported.

Recurrent epistaxis has a significant impact on patients’ quality of life; therefore, treatment is mandatory. Surgical methods could be used locally, but antifibrinolytic and antiangiogenic drugs may also be employed. The effectiveness of therapy can be measured using the Epistaxis Severity Score (ESS) [13]. The management of epistaxis, as recently published guidelines recommend, includes topical treatment, ablative therapy, and antifibrinolytic drugs, such as tranexamic acid. For patients who fail to respond to these treatments, antiangiogenic therapies are also recommended [14].

The diagnosis and treatment of HHT, due to the rarity of the disease, are still challenging. Management of these patients at the University of Debrecen (UD) and surrounding hospitals in Northeast Hungary started almost two decades ago. Our team previously published a review on the Hungarian HHT families and genetic screening [9]. As a continuation of this work, our goal was to provide a summary of the clinical characteristics and treatments used in patients with HHT.

## 2. Materials and Methods

We conducted a retrospective study among HHT patients diagnosed and followed between 2010 and 2025 at the Department of Hematology and the Center of Expertise of Rare Diseases, Institute for Medicine, Faculty of Medicine, University of Debrecen (UD).

The patients’ records were reviewed using UD’s electronic registries (Medsolution and UDMed). Demographic data, clinical presentations, laboratory findings, treatment modalities, and patient outcomes were recorded. Patients who met at least 3 Curacao criteria were included in this study. Telangiectasia in characteristic locations (lips, mouth, fingers) was evaluated by physical examination. Epistaxis severity scores (ESS) were calculated on presentation, and the effect of treatment on ESS was reevaluated 6 months after therapy initiation by two independent physicians. All patients underwent brain, chest, and abdominal computed tomography (CT) scans to assess the AVMs. Patients with blood in their stool underwent upper and lower endoscopies, and, in some cases, a capsule endoscopy to detect small intestinal telangiectasia. An infectologist reviewed patients with pulmonary AVMs or cranial AVMs. Laboratory tests, such as total blood count, serum iron and ferritin levels, transferrin and transferrin saturation levels, and the patient’s genetic findings, were also collected. Desmopression was administered as oral tablets in a 0.1 mg dose for 3 months. Thalidomide was used in a 50 mg oral dose after receiving the drug reimbursement until the occurrence of side effects. Bevacizumab was used in a 5 mg/kg dose every 4th week, receiving the drug reimbursement until the occurrence of side effects. The first infusion was administered 90 min after 40 mg of methylprednisolone premedication. The repeated infusion was administered in 60 min without premedication. Echocardiography and a cardiac check-up are performed annually in patients receiving bevacizumab.

Statistical analysis was performed using IBM SPSS Statistics 27.0. Continuous variables were reported as means and standard deviations. The Kolmogorov–Smirnov test elevated the distributions. We used the χ^2^ and Fisher’s exact test to determine differences between cohorts for categorical variables. We used the Mann–Whitney U test to assess discrepancies between two groups of continuous nonparametric variables and the Kruskal–Wallis test for three groups. The estimation of the effect size of treatment on the ESS values was measured using Cohen’s d, where a value of 0.2 indicates a small effect size, 0.5 a medium, and 0.8 a large effect size. Changes in ESS values were also evaluated using a paired-samples Wilcoxon signed-rank test. All *p*-values were two-tailed with a significance of 0.05 to detect statistical significance.

The retrospective chart review was done after obtaining approval from the Regional and Institutional Ethics Committee of the University of Debrecen (RKEB.IKEB 6836-2024).

## 3. Results

### 3.1. Demographic Data

**Figure 1:**
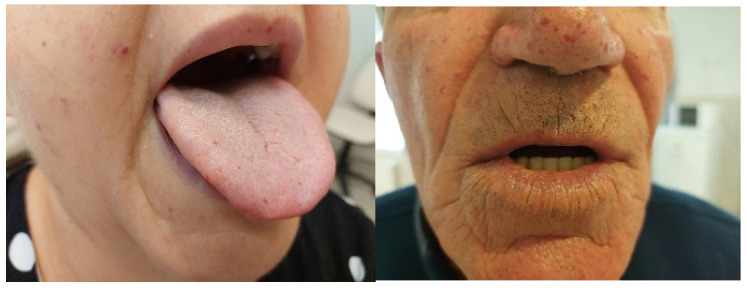
Telangiectasia in characteristic locations.

Forty-eight patients (21 men and 27 women) with HHT followed for 15 years (2010–2025) at the Department of Internal Medicine, Division of Hematology, and Center for Rare Diseases were included in this study. All patients underwent a detailed internal and ENT physical examination. The mean age of the patients was 54 ± 15 years; the difference in age between the genders was not significant (57.2 ± 13.92 for males and 50.42 ± 14.61 for females, *p* = 0.12). Most patients presented with epistaxis, with a mean ESS before treatment of 3.25 ± 1.99 [95% CI, Cohen’s d value: 0.146]; however, the difference between genders was not statistically significant (*p* = 0.69). Fifteen percent of females (n = 4) had severe nosebleeds (ESS > 6), 22% (n = 6) moderate (ESS 4–6), and 63% (n = 17) had mild bleeding, with an ESS score of less than 3. Fourteen percent of males (n = 3) experienced severe epistaxis, and an additional 14% (n = 3) had moderate epistaxis, while 72% (n = 15) showed mild bleeding. All patients had telangiectasia at typical locations (Figure 1). Thirty patients (62%) had an ENG mutation and thus had the HHT1 phenotype; most had pulmonary AVMs, with some having cerebral and hepatic AVMs. Data of subgroups are presented in Table 1. The diagnosis of cerebral AVM was usually made by screening, and in two patients, both of whom had pulmonary AVMs, the presenting symptoms were headache and seizures. Both of them had brain abscesses and were subjected to brain surgery, followed by prolonged antibiotic treatment. Two patients out of 6 in the HHT1 group had cerebral AVM without bleeding or abscess; one patient, also having PAVM, had an ischemic stroke and cerebral complications as a thrombotic event.

### 3.2. Treatment of the Patients

Most patients were referred to our center of reference by an ENT specialist, a general practitioner, or relatives familiar with HHT. These patients already had a history of using topical treatment or sclerotherapy. Oral tranexamic acid was administered to 22 patients to reduce bleeding 2 or 3 times a day (maximum dose was 4 g). Desmopressin acetate (DDAVP), a synthetic analogue of antidiuretic hormone (ADH), was administered to seven patients with ESS > 3 for 3 months, with a moderate effect. Based on hemoglobin and serum iron levels, we recommended iron replacement. In severe iron deficiency, we used intravenous iron supplementation every three months. Blood transfusions were also used in critical cases, usually 4–6 units/year.

In patients who failed to respond to the therapies mentioned above, antiangiogenic treatment was recommended for those with ESS > 3 and severe, daily occurring epistaxis causing transfusion-dependent anemia. Oral thalidomide at a 50 mg/day dose was initiated in 24 patients. Still, because of the known neurological side effects of the treatment, we tried to reduce the dose to the minimum effective level (see later). Twelve patients (40%) had the HHT1 phenotype (40% of the entire cohort), while 12 patients had HHT2 (62% of the whole HHT2 cohort). Treatment data of the two subgroups are provided in Table 2.

Patients with *ACVRL1* mutation were more prone to developing severe bleeding; thus, the use of antiangiogenic treatment was more frequent in this group. In the HHT1 group, thalidomide use lasted 31.08 ± 22.37 months, with the most extended treatment duration reaching 7 years (84 months). This treatment modality was usually well tolerated. In patients who showed significant improvement in bleeding and reduced transfusion dependency, we have reduced the frequency of thalidomide to every other day. The treatment resulted in a considerable decrease in ESS after 6 months (ESS: 2.62 ± 0.99; [95% CI, Cohen’s d value: 0.426 (*p* = 0.699)]). Side effects of thalidomide are not common in such a small dose of therapy, but peripheral neuropathy could occur; thus, use of thalidomide was stopped in those patients reporting numbness. In these cases, electroneurography (ENG) was performed. In the HHT1 and HHT2 groups, 3 patients had severe peripheral neuropathy verified by ENG. Thalidomide was discontinued in 3 patients with *ENG* mutation. Still, because of having a high ESS (average 7.14), severe gastrointestinal bleeding, epistaxis, and transfusion dependency, bevacizumab treatment was initiated at a 5 mg/kg dose every fourth week intravenously. In the HHT1 group, bevacizumab was used for 27.33 ± 24.54 months, further reducing the ESS to 1.45; patients had experienced only rare episodes of epistaxis, and their hemoglobin, serum iron, and ferritin levels improved. Baseline and after treatment laboratory parameters are provided in Table 3.

HHT1 patients received an average of 3.8 units of blood per year, which decreased to 1 unit/year in those whose treatment was less effective; however, the need for transfusion ceased in those who responded well to treatment (the small sample size was not suitable for a rigorous statistical analysis).

**Figure 2:**
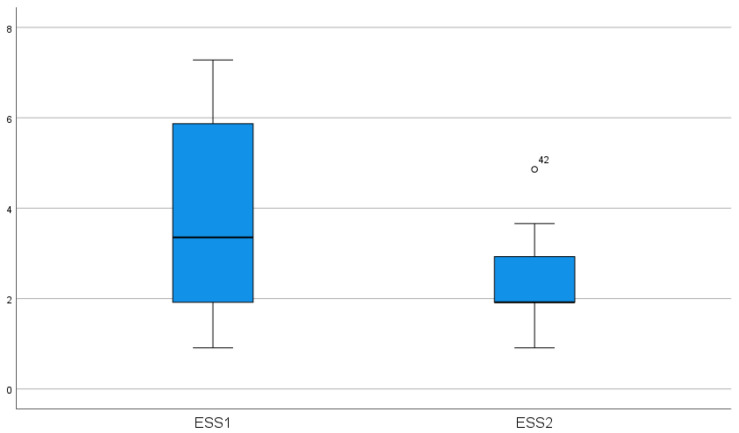
Effect of thalidomide treatment on ESS (*p* < 0.001) (ESS1-epistaxis score before treatment, ESS-2 epistaxis severity score after treatment).

In the HHT2 group, bevacizumab was started in two patients who developed peripheral neuropathy beyond using thalidomide. The average ESS for these patients was 7.73; therefore, bevacizumab was initiated at the dose mentioned earlier. One patient experienced significant improvements in quality of life, and transfusion dependency ceased. In contrast, a 50-year-old woman still had very severe epistaxis requiring transfusions every month. The number of bevacizumab-treated cases is too small to perform a statistical analysis. Using the Wilcoxon signed-rank test for paired ESS, we found a significant reduction in ESS in both subgroups (HHT1: *p* = 0.003; HHT2: *p* = 0.043). Thalidomide treatment caused a significant decrease in ESS in both groups (*p* < 0.001) (Figure 2).

**Figure 3:**
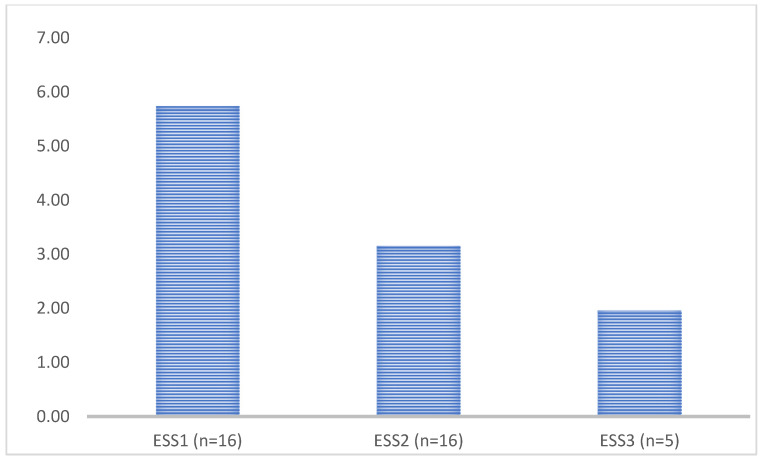
Epistaxis severity score (ESS) of patients after antiangiogenic treatment (ESS1 baseline data of patients with moderate ESS > 3, ESS2 6 months after thalidomide treatment, ESS3 6 months after bevacizumab treatment).

Bevacizumab treatment further reduced the ESS, but the few cases were not suitable for statistical analysis; however, the trend of decrease suggests that the treatment is more effective (Figure 3).

### 3.3. Case Report

**Figure 4:**
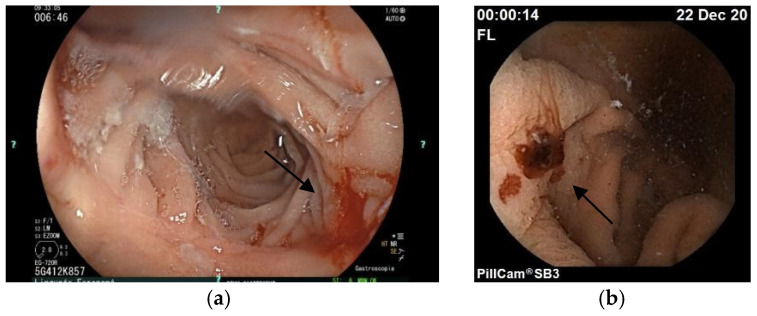
(**a**) The arrow shows a telangiectasia in the duodenum by gastroscopy, (**b**) the arrow shows gastric telangiectasia by capsule endoscopy.

We present the case of a 50-year-old woman in whom the difficulties of treatment in HHT are evident. The HHT was diagnosed in 2009, based on severe epistaxis, telangiectasia on characteristic sites, gastrointestinal bleeding due to gastric and duodenal telangiectasia, and finally, a positive family history of HHT. She had severe iron-deficiency anemia; thus, we recommended iron replacement orally, and every 3 months intravenously, and DDAVP before each invasive procedure. Chest and brain CTs were done, which did not reveal an AVM. Genetic testing found a new pathogen, a heterozygote mutation in the *ACVRL1* gene (c.997A>T, p.Ser333Cys). She needed ENT care and hospitalization almost every month due to severe transfusion-dependent anemia. Argon plasma treatment was also regularly done in gastroenterology. DDAVP nasal spray and tranexamic acid (2 × 500 mg), an antifibrinolytic drug, were initiated. In July of 2020, oral DDAVP (0.1 mg) was recommended every second day at the individual’s request. However, she was still bleeding regularly. In January 2021, thalidomide was started at a 50 mg dose, based on previous case reports. Since thalidomide is an off-label treatment in Hungary and not reimbursed, compassionate use is required. In February 2022, the thalidomide dose was increased to 100 mg. Still, after 3 months, she was complaining about numbness in the legs, ENG showed sensomotoric neuropathy, vitamin B complex was started, and thalidomide ceased. The clinical symptoms had not improved, so we obtained permission to use bevacizumab and initiated treatment at a dose of 5 mg/kg in December 2022. She received eight infusions every 3 weeks. However, this treatment did not improve the gastrointestinal bleeding; she regularly needed gastroscopy, laser coagulation, and, lastly, an Ovesco (over the scope) clip to stop severe bleeding was used (Figure 4).

All routine hemostatic tests (PT: 7.6 s, INR 0.92, APTT 36.7 s, TT 13.7 s, fibrinogen 3.71 g/L), platelet count, aggregometry (PFA-platelet function analysis: collagen/adrenaline 93 s) were normal. Other tests, such as von Willebrand (VWF) factor (103 g/L) and vWF: Ristocetin cofactor activity, were 160% of the regular level; FVIII activity was 95%. Share wave elastography (Aixplorer) showed a grade 2 liver cirrhosis, abdominal ultrasound revealed hepatic steatosis, AVM was not detected, and a mildly dilated central bile duct was shown without a stone. Liver enzymes were only slightly elevated (Aspartate aminotransferase-AST: 25 U/L, Alanine aminotransferase-ALT: 20 U/L, gamma-glutamyl transferase-GGT: 142 U/L, alkaline phosphatase-ALP: 273 U/L, total protein-TP: 52 g/L, albumin-Alb: 33 g/L, total bilirubine: 3.7 umol/L). Wall motion abnormality and valvulopathy were excluded by transthoracic echocardiography; the right ventricular pressure was normal (30 + 3 mmHg). The incipient liver cirrhosis may have caused portal hypertension, increasing the bleeding tendency too. A non-selective beta-blocker (propranolol) and a mineralocorticoid receptor antagonist were started.

**Figure 5:**
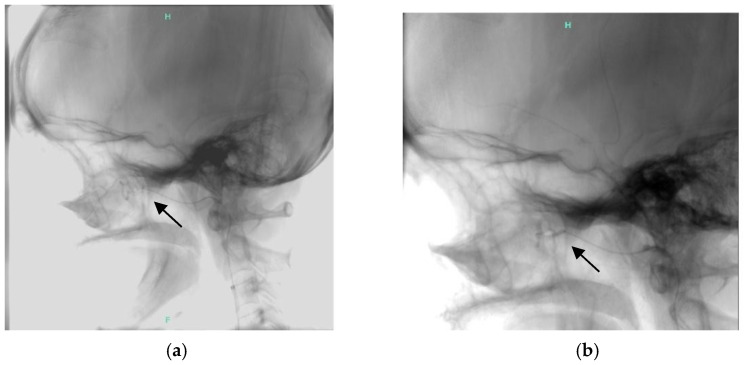
Superselective embolisation of the internal maxillary artery (arrow shows (**a**) the end of the catheter showing AVM, (**b**) after embolisation).

Finally, our team decided on a gastric resection; on 28 June 2023, a subtotal gastrectomy with Roux-Y reconstruction was performed laparoscopically in the Department of Surgery. After this procedure, she still had gastrointestinal bleeding and epistaxis; thus, we restarted bevacizumab treatment in August 2024, at a 5 mg/kg dose every 6th weeks. Beyond this, she had been hospitalized regularly because of severe epistaxis to the ENT and received transfusions. Finally, we decided to recommend intervention, and superselective embolization of the internal maxillary arteries on both sides was done successfully (Figure 5).

## 4. Discussion

Osler-Weber-Rendu disease is a rare vascular disorder with autosomal dominant inheritance. The diagnosis and treatment, as well as patients’ follow-up, require a multidisciplinary approach. In this study, we present our 15 years of experience in diagnosing and treating these patients. The diagnosis is based on the Curacao criteria [6]; if three or more criteria are present, the diagnosis is definite.

The typical age of disease presentation is adolescence or adulthood. Recurrent nosebleeds usually start between the ages of 12 and 16. Seventy-one percent of patients will develop symptoms by age 40, and more than 90% of patients will have a clinical presentation [15]. Beyond pulmonary and cerebral AVMs, epistaxis is the most serious and sometimes life-threatening symptom of HHT [16]. Treatment options are based on the severity of nosebleeds, quantified by the ESS, which must be calculated for each presentation [17]. In our study, most patients had mild symptoms based on the ESS; 18.75% had moderate (4–6), and 14.5% had severe bleeding (ESS > 6). There was no difference between genders. According to guidelines, antiangiogenic treatment is recommended in severe cases; however, our experience suggests that other factors also influence the decision. The ESS is an excellent score to assess the activity of a nosebleed. Still, it does not account for gastrointestinal telangiectasia or bleeding originating from the gastrointestinal tract. Hypertension, or portal hypertension, is a risk factor for bleeding, too. Thus, the patient’s quality of life, transfusion-dependency, gastrointestinal bleeding, and uncontrolled blood pressure must be considered when we decide to start antiangiogenic treatment. Desmopressin acetate, a synthetic analog of ADH, was used in our HHT patients based on our previous experience in reducing bleeding in von Willebrand disease. However, it is not recommended treatment in HHT, and it was used in only one case report previously to treat a severe gastrointestinal bleeding of a HHT patient [18]. The mechanism of action is to reduce bleeding by inducing exocytosis of von Willebrand factor and factor VIII from their storage sites in endothelial cells, as well as from Weibel-Palade bodies and platelet alpha granules [19]. Antiangiogenic treatment, such as thalidomide, has been used in the treatment of HHT since 2015, when the drug’s effectiveness was reported in a non-randomized single-center study [20]. In these preliminary studies, the authors administered thalidomide at 50–100 mg/day, resulting in a significant reduction in epistaxis [21]. Pomalidomide was also used in some reports (the PATH-HHT study) [22], but lenalidomide was not used previously. The PAT-HHT study was the largest and best-powered randomized trial; it lasted 24 weeks, whereas our study lasted 27 months. The mean baseline ESS was higher than in our study. We selected 11 patients with mild symptoms, six with moderate symptoms, and seven with severe symptoms who were treated with thalidomide, which significantly reduced the patients’ transfusion dependency and ESS. The treatment was well tolerated; the only detected side effect was peripheral neuropathy in six patients, which resulted in the discontinuation of the therapy. The duration of thalidomide treatment was 29 months, but four patients have been receiving thalidomide without side effects for 48 months. In previous studies, the treatment duration was usually 12 months; only one case report administered the drug for 29 months [23]. Unless peripheral neuropathy, other side effects, such as venous thromboembolism, have not been observed. We also used thalidomide for a 70-year-old male patient with an *ACVRL1* mutation, who previously did not have severe epistaxis unless having a myocardial infarction. Percutaneous cardiac catheterization was done, and a double thrombocyte aggregation inhibitor (AAI) was initiated and recommended for 6 months; as a consequence, severe epistaxis occurred, causing transfusion-dependent anemia. In his case, treatment with thalidomide made it possible to tolerate the AAI.

Tranexamic acid is a beneficial adjunctive treatment for patients with HHT. Fernandez et al. demonstrated that the drug enhances the activity of the ALK-1/endoglin pathways [24] and is effective in treating bleeding [25]. We administered tranexamic acid to 22 HHT patients with non-severe epistaxis or before an invasive procedure, such as a tooth extraction.

Bevacizumab, an anti-VEGF antibody, is effective in treating bleeding and anemia in HHT [26,27,28,29], while nasal bevacizumab did not have a significant impact on controlling nasal bleeding [30]. In previous studies, bevacizumab was more frequently used in patients with *ACVRL1* mutations; in our patients, it was also more frequent in the HHT2 group (3/18 vs. 3/30 in the HHT1 group). We used bevacizumab treatment at a 5 mg/kg dose in six patients who did not tolerate the thalidomide treatment. Because of the low number of cases, adequate statistical analysis was not done, but we detected a significant reduction in ESS and a significant improvement in patients’ quality of life. No complications, such as infections or abnormal wound healing, were observed. In those cases where symptoms improved, we decreased the dosing interval to every 6 weeks. Cardiologic check-ups were done regularly. The maximum treatment duration was 48 months. None of the treatments mentioned above was successful in the 50-year-old female patient presented briefly; in her case, the bleeding tendency is also increased because of portal hypertension; in her case, superselective embolization of the internal maxillary artery was performed successfully. Catheter-mediated embolization is a treatment option for HHT patients, both for nosebleeds, when prior conservative treatment has been unsuccessful. Still, we must consider the serious complications that could occur [31].

## 5. Conclusions

This is the first case series to describe the clinical characteristics and therapeutic challenges in the treatment of HHT patients in Hungary. The management of HHT patients is complex; a multidisciplinary approach is required, and specific permissions must be obtained for the use of both thalidomide and bevacizumab.

We recognize our study’s limitations: it was a retrospective single-center review of a rare disease with a limited sample size, and only moderate-to-severe cases were prioritized for antiangiogenic therapy. The small sample size was insufficient for a serious statistical analysis. Multicenter data with long-term follow-up can improve our results.

Based on our experience, we recommend thalidomide or other anti-angiogenic treatments not only for severe cases but also for those with mild or moderate symptoms, as this approach can help prevent complications and progression.

## Figures and Tables

**Table 1:** Characteristics of patients with HHT1 and HHT2 phenotypes (ESS: epistaxis severity score; GI: gastrointestinal; PAVM: pulmonary AVM; CAVM: cerebral AVM; HAVM: hepatic AVM; NS: non-significant).

	HHT1	HHT2	*p*
n	30	18	
Age (years)	56.29 ± 14.58	48.63 ± 13.57	0.08
Gender			
Male n (%)	15 (50)	6 (33)	
Female n (%)	15 (50)	12 (67)	
ESS before treatment	3.42 ± 1.99	3.00 ± 2.78	
ESS after treatment	2.62 ± 0.99	1.92 ± 1.84	NS
GI telangiectasia n (%)	3 (10)	2	
PAVM (%)	19 (63)	0	
CAVM (%)	6 (20)	1 (6)	
HAVM (%)	6 (20)	6 (33)	
Thalidomide treatment n (%)	12 (40)	12 (67)	
Bevacizumab n (%)	3	2	

**Table 2:** Characteristics of patients receiving antiangiogenic treatment.

	HHT1	HHT2	*p*
Age (years)	56.29 ± 14.58	48.63 ± 0.08	
Gender			
Male (%)	61 (n = 7)	50 (n = 6)	
Female (%)	39 (n = 5)	50 (n = 6)	
Thalidomid n =	12	12	
Duration of treatment (months)	31.08 ± 22.37	27 ± 24.94	NS
Bevacizumab n =	3	2	
Duration of treatment (months)	27.33 ± 24,5	27.33 ± 24.84	NS
ESS before treatment	4.15 ± 1.91	3.79 ± 3.19	
ESS after treatment	2.62 ± 0.99	2.02 ± 1.91	NS

**Table 3:** Laboratory data of patients before and after antiangiogenic treatment.

	HHT1	*p*	HHT2	*p*
Iron level I. (N: 6.6–26 μmol/L)	5.88 ± 3.59	0.06	6.22 ± 4.33	0.01
Iron level II. (N: 6.6–26 μmol/L)	13.93 ± 42.84		15.59 ± 6.68	
Ferritin I. (N: 15–150 μg/L)	42.83 ± 52.18	0.54	24.56 ± 52.59	0.07
Ferritin II. (N: 15–150 μg/L)	52.18 ± 37.53		20.17 ± 33.4	
TFR saturation I. (N: 16.0–45.0%)	9.05 ± 6.73	0.2	8.13 ± 4.64	0.04
TFR saturation II. (N: 16.0–45.0%)	15.45 ± 12.7		19.52 ± 10.87	
Hemoglobin I. (g/L)	118.44 ± 24.36	0.69	104.25 ± 15.12	0.41
Hemoglobin II. (g/L)	122.55 ± 27.20		117.38 ± 29.31	

## Data Availability

The data presented in this study are available on request from the corresponding author. The data are not publicly available due to privacy and ethical considerations.

## Abbreviations

The following abbreviations are used in this manuscript:

AAI	antiplatelet aggregation inhibitor
*ACVRL1*	activin receptor-like kinase 1
ADH	antidiuretic hormone
AVM	arteriovenous malformation
BMP	bone morphogenic protein
CT	computed tomography
DDAVP	desmopressin acetate
*ENG*	endoglin
ENG	electroneurography
ENT	ear, nose, and throat specialist
ESS	epistaxis severity score
HHT	hereditary hemorrhagic telangiectasia
Ovesco clip	over the scope clip
PFA	platelet function analysis
TGFβ	transforming growth factor β
UD	University of Debrecen
VEGF	vascular endothelial growth factor
VWF	von Willebrand factor

## References

[1] Fuchizaki U., Miyamori H., Kitagawa S., Kaneko S., Kobayashi K. (2003). Hereditary haemorrhagic telangiectasia (Rendu-Osler-Weber disease). Lancet.

[2] Kritharis A., Al-Samkari H., Kuter D. (2018). Hereditary hemorrhagic telangiectasia: Diagnosis and management from the hematologist’s perspective. Haematologica.

[3] Shovlin C., Guttmacher A., Buscarini E., Faughnan M., Hyland R., Westermann C., Kjeldsen A.D., Plauchu H. (2000). Diagnostic criteria for hereditary hemorrhagic telangiectasia (Rendu-Osler-Weber syndrome). Am. J. Med. Gen..

[4] Marchuk D. (1998). Genetic abnormalities in hereditary hemorrhagic telangiectasia. Curr. Opin. Hematol..

[5] McDonald M., Bayrak-Toydemir P., DeMille D., Wooderchak-Donahue W., Whitehead K. (2020). Curacao diagnostic criteria for hereditary hemorrhagic telangiectasia is highly predictive of a pathogenic variant in *ENG* or *ACVRL1* (HHT1 and HHT2). Genet. Med..

[6] McDonald M., Bayrak-Taydemir P., Pyeritz R. (2011). Hereditary hemorrhagic telangiectasia: An overview of diagnosis, management, and pathogenesis. Genet. Med..

[7] Cerda P., Castillo S., Aguilera C., Iriarte A., Rocamora J., Larrinaga A., Vinals F., Graupera M., Riera-Mestre A. (2024). New genetic drivers in hemorrhagic hereditary telangiectasia. Eur. J. Int. Med..

[8] Major T., Gindele R., Szabó Z., Jóni N., Kis Z., Bora L., Bárdossy P., Rácz T., Karosi T., Bereczky Z. (2019). Genetic diagnostics of hereditary hemorrhagic telangiectasia (Osler-Webere-rendu disease). Orv. Hetil..

[9] Major T., Bereczky Z., Gindele R., Balogh G., Rácz B., Bora L., Kézsmárki Z., Brúgós B., Pfliegler G. (2021). Current status of clinical and genetic screening of hereditary hemorrhagic telangiectasia families in Hungary. J. Clin. Med..

[10] Cole S., Begbie M., Wallace G., Shovlin C. (2005). A new locus for hereditary hemorrhagic teleangiecatsia (HHT3) maps to chromosome 5. J. Med. Genet..

[11] Bayrak-Taydemir P., McDonald J., Akarsu N. (2006). A fourth locus for hereditary hemorrhagic telangiectasia maps to chromosome 7. Am. J. Med. Genet. A.

[12] Hernandez F., Huether R., Carter L. (2015). Mutations in RASA1 and GDF2 were identified in patients with clinical features of hereditary hemorrhagic telangiectasia. Hum. Genom. Var..

[13] Hammill A., Wusik K., Kasthuri R. (2021). Hereditary hemorrhagic telangiectasia (HHT): A practical guide to management. Hematol. Am. Soc. Hematol. Educ. Program.

[14] Faughnan M., Mager J., Hetts S., Palda V., Lang-Robertson K., Buscarini E., Deslandres E., Kasthuri R.S., Lausman A., Poetker D. (2020). Second International Guidelines for the Diagnosis and Management of Hereditary Hemorrhagic TelangiecTASIA. Ann. Intern. Med..

[15] Begbie M., Wallace G., Shovlin C. (2003). Hereditary haemorrhagic telangiectasia (Osler-Weber-Rendu syndrome): A view from the 21st century. Postgrad. Med. J..

[16] Rebeiz E., Bryan D., Ehrlichman R., Shapshay S. (1995). Surgical management of life-threatening epistaxis in Osler-Weber-Rendu disease. Ann. Plast. Surg..

[17] Hoag J., Terry P., Mitchell S., Reh D., Merlo C. (2010). An epistaxis severity score for hereditary hemorrhagic telangiectasia. Laryngoscope.

[18] Quitt M., Froom P., Veisler A., Falber V., Sova J., Aghai E. (1990). The effect of desmopressin on massive gastrointestinal bleeding in hereditary telangiectasia unresponsive to treatment with cryoprecipitate. Arch. Intern. Med..

[19] Özgönenel B., Rajpurkar M., Lusher J.M. (2007). How do you treat bleeding disorders with desmopressin?. Postgrad. Med. J..

[20] Invernizzi R., Quaglia F., Klersy C., Pagella F., Ornati F., Chu F., Matti E., Spinozzi G., Plumittalo S., Grignani P. (2015). Efficacy and safety of thalidomide for the treatment of severe recurrent epistaxis in hereditary haemorrhagic telangiectasia: Results of a non-randomised, single-centre, phase 2 study. Lancet Haematol..

[21] Fang J., Chen X., Zhu B., Ye H., Zhang W., Guan J., Kaiming S. (2017). Thalidomide for epistaxis in patients with hereditary hemorrhagic telangiectasia: A preliminary study. Otolaryngol. Head Neck Surg..

[22] Al-Samkari H., Kasthuri R., Iyer V., Pishko A., Decker J., Whitehead K., Conrad M.B., Weiss C., Parambil J., Zumberg M.S. (2023). PATH-HHT, a double-blind, randomized, placebo-controlled trial in hereditary hemorrhagic telangiectasia, demonstrates that pomalidomide reduces epistaxis and improves quality of life. Blood.

[23] Ugur M., Baysal M., Umit E. (2024). The role of thalidomide and its analogs in the treatment of hereditary hemorrhagic telangiectasia: A systemic review. J. Clin. Med..

[24] Fernandez-L A., Garrido-Martin E.A., Sans-Rodriguez F., Ramirez J.R., Morales-Angulo C., Zarrabeitia R., Perez-Molino A., Bernabéu C., Botella L.-M. (2007). Therapeutic action of tranexamic acid in hereditary haemorrhagic telangiectasia (HHT): Regulation of ALK-1/endoglin pathway in endothelial cells. Thromb. Haemost..

[25] Zaffar N., Ravichakaravarthy T., Faughnan M., Shehata N. (2014). The use of anti-fibrinolytic agents in patients with HHT: A retrospective survey. Am. Hematol..

[26] Al-Samkari H., Kasthuri R., Parambil J., Albitar H., Almodallal Y., Vazquez C., Serra M., Dupuis-Girod S., Wilsen C.B., McWilliams J.P. (2021). An international, multicenter study of intravenous bevacizumab for bleeding in hereditary hemorrhagic telangiectasia: The InHIBIT-bleed study. Haematologica.

[27] Bose P., Holter J., Selby G. (2009). Bevacizumab in hereditary hemorrhagic telangiectasia. N. Engl. J. Med..

[28] Epperla N., Kapke J., Karafin M., Friedman K., Foy P. (2016). Effect of systemic bevacizumab in severe hereditary hemorrhagic telangiectasia associated with bleeding. Am. J. Hematol..

[29] Dupuis-Girod S., Riviere S., Lavigne C., Fargeton A., Guilbert-Dussardier B., Grobost V., Leguy-Seguin V., Maillard H., Mohamed S., Decullier E. (2023). Efficacy and safety of intravenous bevacizumab on severe bleeding associated with hemorrhagic telangiectasia: A national randomized multicenter trial. J. Int. Med..

[30] Stokes P., Rimmer J. (2018). Intranasal bevacizumab in the treatment of HHT-related epistaxis: A systematic review. Rhinology.

[31] Sobrepera S., Monroe E., Gemmete J., Hallam D., Pinchot J., Kaufman C. (2021). Imaging to intervention: A review of what the interventionalist needs to know about hereditary hemorrhagic telangiectasia. CVIR Endovas..

